# *Ndae1* Expression and Regulation in *Drosophila* Embryos

**DOI:** 10.1371/journal.pone.0092956

**Published:** 2014-03-27

**Authors:** Maria Florencia Tevy, Denis Seyres, Concetta Traina, Laurent Perrin, Maria Capovilla

**Affiliations:** 1 Dulbecco Telethon Institute, Department of Life Sciences and Biotechnology, University of Ferrara, Ferrara, Italy; 2 INSERM, UMR 1090 TAGC, Marseille, France; 3 Aix-Marseille Université, UMR1090 TAGC, Marseille, France; 4 CNRS, Marseille, France; 5 Institut Sophia Agrobiotech, UMR 7254 INRA/CNRS/UNS, Sophia Antipolis, France; University of Florida, United States of America

## Abstract

The construction and prediction of cell fate maps at the whole embryo level require the establishment of an accurate atlas of gene expression patterns throughout development and the identification of the corresponding *cis*-regulatory sequences. However, while the expression and regulation of genes encoding upstream developmental regulators such as transcription factors or signaling pathway components have been analyzed in detail, up to date the number of *cis*-regulatory sequences identified for downstream effector genes, like ion channels, pumps and exchangers, is very low. The control and regulation of ion homeostasis in each cell, including at blastoderm stages, are essential for normal embryonic development. In this study, we analyzed in detail the embryonic expression pattern and *cis*-regulatory modules of the *Drosophila Na^+^-driven anion exchanger 1* (*Ndae1*) gene, involved in the regulation of pH homeostasis. We show that *Ndae1* is expressed in a tight and complex spatial-temporal pattern. In particular, we report that this downstream effector gene is under the control of the canonical dorsal-ventral patterning cascade through *dorsal*, *Toll*, *twist* and *snail* at early embryogenesis. Moreover, we identify several *cis*-regulatory modules, some of which control discrete and non-overlapping aspects of endogenous gene expression throughout development.

## Introduction

Throughout embryogenesis, morphogen gradients and signaling molecules lead to a precise combination in the spatial distribution of gene expression. During this process, effector genes are switched on and off through their *cis*-regulatory sequences in a highly dynamic manner to grant cell fate and achieve a specific developmental program. For the construction, prediction and validation of cell fate maps at the whole embryo level, a main issue is to acquire an accurate atlas of gene expression patterns and their corresponding *cis*-regulatory sequences [1]–[3]. In addition, one of the major challenges of today embryology is to infer spatio-temporal *cis*-regulatory activity based on *in vivo* transcription factor binding and enhancer activity data. In *Drosophila*, numerous methods to computationally predict or validate *cis*-regulatory sequences at a genome wide scale have been developed [2],[4]–[6]. Despite these many efforts, the number of *cis*-regulatory sequences of downstream effector genes is very low. Hence, in this era of genomics and high throughput analysis, traditional methods are still useful to complement and refine findings, adding accuracy to databases for the community.

Among the downstream effector genes whose regulatory network is yet to be unraveled are ion channels, pumps and exchangers. Normal embryonic development depends on the control of essential functions like ion homeostasis and the inability to maintain ion homeostasis leads to severe developmental defects [7] and diseases, called channelopathies [8], [9]. However, there are still few studies on how morphogen gradients and master regulators affect ion homeostasis in *Drosophila* embryogenesis. One such study reveals how calcium gradients are required for specification of the dorsal embryonic region and how this gradient is inhibited in *pipe, Toll* (*Tl*), and *dorsal* (*dl*) mutants, but is unchanged in *decapentaplegic* or *punt* mutants. At stage 5 of *Drosophila* embryonic development a calcium gradient is formed and is used for patterning the embryo through the suppression of ventral calcium concentrations [10], [11]. Ion channels, pumps and exchangers are also useful markers of cell fate determination, as it has been observed during *Drosophila* neuronal specification. Indeed, different neurons are specified by different sets of TFs activating different sets of ion channels, which in turn confer distinct electrophysiological properties to each particular type of neuron [12], [13].

*Drosophila Ndae1* encodes a membrane-associated protein of 1,030 aminoacids, with homology to the vertebrate family of Na^+^-dependent Cl^−^/HCO_3_^−^ exchangers [14]. When expressed in *Xenopus* oocytes, *Ndae1* mediates the exchange of Na^+^, Cl^−^ and HCO independently of HCO_3_^−^, in order to maintain intracellular hydrogen ion concentration (pH) [14]–[16]. The regulation of pH is fundamental for cell viability, metabolism and action potentials [15], [17].

In *Drosophila* embryos, *Ndae1* expression has been detected at blastoderm stages, and later in the alimentary tract, Malpighian tubules, salivary glands, mesectoderm, anal pads and nervous system [14], [18]. Altogether these data reinforce the notion that *Ndae1* may be a key pH and osmoregulatory protein during early development and may contribute to baso-lateral ion transport in *Drosophila* epithelia and in the nervous system. Hence, *Ndae1* expression must be under tight dynamic control throughout development and therefore constitutes an interesting candidate to study its regulation.

In vertebrates, Na^+^-dependent Cl^−^/HCO_3_^−^ exchangers are a very extensive gene family constituted by a large number of proteins and isoforms [16], [19], [20]. Like *Drosophila Ndae1*, many members of this family are widely expressed. Among their functions, is the maintenance of intracellular pH. The importance of these channel functions is illustrated by studies in knock out models and the human diseases to which they have been linked (reviewed in [16], [20]). For example, SLC4A4 has been demonstrated to be expressed in the colon and kidneys and knock out mice (NBCe1^-/-^) show metabolic acidosis and intestinal blockage ([21] and references therein). Further, members of this family of transporters, like *Drosophila Ndae1*, are expressed in the brain, though the differential expression in neuronal and glial subtypes still requires further investigation. Because neuronal activity may provoke changes in pH and localized changes in pH may in turn trigger neuronal activity [22], the functions of these channels in the brain is crucial for the adequate functioning of network excitability. Indeed, the Na^+^-driven Cl^−^/HCO_3_^−^ exchanger Slc4a8 was shown to be localized to presynaptic terminals of glutamatergic neurons and hippocampal cultured neurons of knock out mice show impaired glutamate release that can be alleviated by increasing intracellular pH [23]. Likewise, Slc4a10 null mice show reduced neuronal excitability [24]. Importantly, SLC4A10 has been found to be mutated in patients with epilepsy [25], [26] and has been associated to autism spectrum disorders [27]. These growing body of evidence points out to a prominent role of Na^+^-driven Cl^−^/HCO_3_^−^ exchangers and *Ndae1* in network excitability. More and more, evidence suggests that alterations in network excitability not only drive brain plasticity mechanisms, but also constitute the early molecular preclinical symptom of many epilepsies, psychiatric disorders and neurodegenerative diseases like Alzheimer disease or Parkinson [28]–[30]. Considering the high complexity of vertebrate models and in particular their high genetic redundancy, alternative models are required for the study of the functions and regulation of bicarbonate transporters.

Here, we accurately analyze *Ndae1* expression in embryos using a highly sensitive *in* situ hybridization technique. We report *Ndae1* as one of the few downstream effector genes under the control of the classical dorsal-ventral (DV) patterning cascade acting through *twist* (*twi*) and *snail* (*sna*). Moreover, we show the analysis of several *Ndae1 cis*-regulatory sequences and demonstrate that the complex pattern of *Ndae1* spatio-temporal expression is driven by multiple enhancers.

## Materials and Methods

### Transformation constructs

All DNA fragments have been amplified by PCR using the KOD or KOD XL (when greater than 3,000 bp) DNA Polymerase (Novagen, Toyobo). The following constructs (except for pMC035) have been cloned from a *w^1118^* genomic DNA template into the P{CaSpeR-hs43-AUG-betagal} (CHAB) vector (V. Pirrotta, unpublished data) in their endogenous orientation. In the oligonucleotides, the linkers are shown in bold and the restriction sites are underlined. pMC008: 2,400 bp corresponding to intron I amplified with the primers FWD 5′-**GG** GCGGCCGCCTGAAAAGAAGAGTGGCATT-3′ (oMC007) and REV 5′-**GG** GGATCCGTAAGTAGGACGGTGTGATT-3′ (oMC008) and cloned into NotI/BamHI. pMC013: a 4,000 bp BamHI-Xba I fragment corresponding to the most 5′ fragment of the 5′UTR was CRE excised from a lambda PS library, subcloned in BamHI/XbaI of pBluescript KS+, excised with BamHI and NotI and cloned into CHAB in BamHI/NotI. pMC020: 963 bp corresponding to the complete 3′UTR of *Ndae1* amplified with the primers FWD 5′-**CCG** CTCGAGCTTCAATATAAAATGGCATATTTGCA-3′ (oMC031) and REV 5′-**CG** GAATTCAAAATCCCACGGACCAGTG-3′ (oMC032) and cloned into XhoI/EcoRI. pMC024: 1,353 bp corresponding to introns VIII and IX in addition to exon IX amplified with the primers FWD 5′-**TTGCTC** GAGCTGTAATATAAACCATTGGGTACAGTT-3′ (oMC033) and REV 5′-**CCG** GAATTCGTGAGTCGAATAAATCAATTAAAACAA-3′ (oMC034) and cloned into XhoI/EcoRI. pMC025: 2,628 bp corresponding to a fragment of the 5′UTR overlapping with pMC027 and pMC029 amplified with the primers FWD 5′-GGATCCGGTGTGCTAATCAAGTTTACGTCG-3′ (oMC013) and REV 5′-CCGCGGACAACAGGGCGTATGAATTCG-3′ (oMC014) and cloned into BamHI/SacII. pMC026: 2,689 bp corresponding to introns II and III amplified with the primers FWD 5′-**GG** CTCGAGGTT**C**AAGGATACTTTATGAGAAACAGA-3′ (oMC037) and REV 5′-**GGG** GAATTCGTGAGTCGAGCTTCACTTTTTAAAG-3′ (oMC038) and cloned into XhoI/EcoRI. pMC027: 4,600 bp corresponding to a fragment of the 5′UTR amplified with the primers FWD 5′-**CG** GGATCCCAACCCAACACCCCTTCA-3′ (oMC017#2) and REV 5′-**AT** CCGCGGCCCAAAGTGGACATGCAG-3′ (oMC018#2) and cloned into BamHI/SacII. pMC028: 3,343 bp corresponding to intron III amplified with the primers FWD 5′-**CCG** CTCGAGCTGCATTAGTCGCGTTTTTTT-3′ (oMC035) and REV 5′-**TTTTT** GCGGCCGCGTGAGTATGGGGGTGTTCAC-3′ (oMC036) and cloned into XhoI/NotI. pMC029: 4,272 bp corresponding to a fragment of the 5′UTR amplified with the primers FWD 5′-**CG** GGATCCGCCGATCTTTAAACTGAAGCA-3′ (oMC015#2) and REV5′- **AT** CCGCGGGACAACAGGGCGTATGAATTC-3′ (oMC016#2) and cloned into BamHI/SacII. pMC035: 3,343 bp corresponding to intron IV amplified from the RP9816C17 BAC DNA with the primers FWD 5′-**CC** GCTAGCCTGCATTAGTCGCGTTTTTTT-3′ (oMC057) and REV 5′-**CC** AGATCTTGAGTATGGGGGTGTTCAC-3′ (oMC060) and cloned into NheI/Bglll of the pH-Stinger vector [31].

### Fly strains

*dl^1^* (BL#3236), *dl^4^* (BL#7096), Tl^10B^ (BL# 30914), *twi^1^* (BL#2381), *sna^18^* (BL#2311), *twi^3^*, *sna^18^* (BL#3299) mutants and *w^1118^* (BL#5905) flies were acquired from the Bloomington Drosophila Stock Center (Bloomington, IN, USA). Because the *dl^1^* and *dl^4^* stocks resulted homozygous lethal, transheterozygote *dl^1^*/*dl^4^* females were generated and used to lay eggs after crossing to *w^1118^* males. All constructs were injected into *w^1118^* embryos by standard procedures to make transgenic flies bearing the same name as the transgene they carry.

### *In situ* hybridization and immunohistochemistry

The *Ndae1* sense probe was synthesized from a full length *Ndae1* cDNA (AF047468) cloned into the pGEM-T easy plasmid [32] linearized with SacII and transcribed with the SP6 RNA polymerase using the Riboprobe Combination System kit (Promega, Madison, WI) and the DIG RNA Labeling Mix (Roche, Indianapolis, IN). This cDNA encompasses the full *Ndae1* coding sequence and therefore allows revealing all characterized *Ndae1* transcripts. The control sense probe was synthesized using the T7 RNA polymerase. The GFP probe was made as previously [33]. Embryos were fixed in 4% formaldehyde for 25 minutes and stored in 100% ethanol. Endogenous peroxidase was inactivated by incubation in 3% H_2_O_2_ for 30 minutes. Then, embryos were post-fixed in PBS containing 8% Triton X-100 as in [34]. Embryos were stained using the Tyramide Signal Amplification (TSA) Plus Biotin System (Perkin Elmer n. NEL700A001KT) following instructions except that SA-HRP (Perkin Elmer n. NEL750001EA) and TSA were diluted at 1∶500. Endogenous biotin was blocked using the Avidin/Biotin Blocking kit (Vectorlab n. SP2001) before adding the biotinylated anti-Digoxigenin antibody. Details of the protocol are available in Text S1. All antibodies were previously pre-absorbed on embryos at a 1∶10 dilution. The anti-Dig antibody (Jackson ImmunoResearch) was used at a 1∶250 dilution. For immunohistochemistry, rabbit anti-β-Galactosidase (Life Technologies) and rabbit anti-GFP (Molecular Probes, Invitrogen) were used at a 1∶500 dilution. Histochemical detection was carried out using biotinylated goat anti-rabbit IgG or biotinylated goat anti-mouse IgG (1∶500 dilution) and the avidin/biotin reagents of the Vectastain Elite Kit (Vector labs). Color was revealed using 0.5 mg/ml 3,3′-Diaminobenzidine tetrahydrochloride hydrate and approximately 0.0015–0.003% H_2_O_2_. For immunofluorescence, chicken anti-GFP (Aves Labs, Inc). rabbit anti-Mef2 (made by BioGenes) and Mab 22C10 (Hybridoma Bank) antibodies were used at a 1∶1,000 dilution. Alexa 488 coupled goat anti-chicken and Alexa 555 coupled goat anti-rabbit secondary antibodies (Molecular Probes, Invitrogen) were used at 1∶500. Slides were observed on a Zeiss LSM 510 confocal microscope.

## Results and Discussion

**Figure 1:**
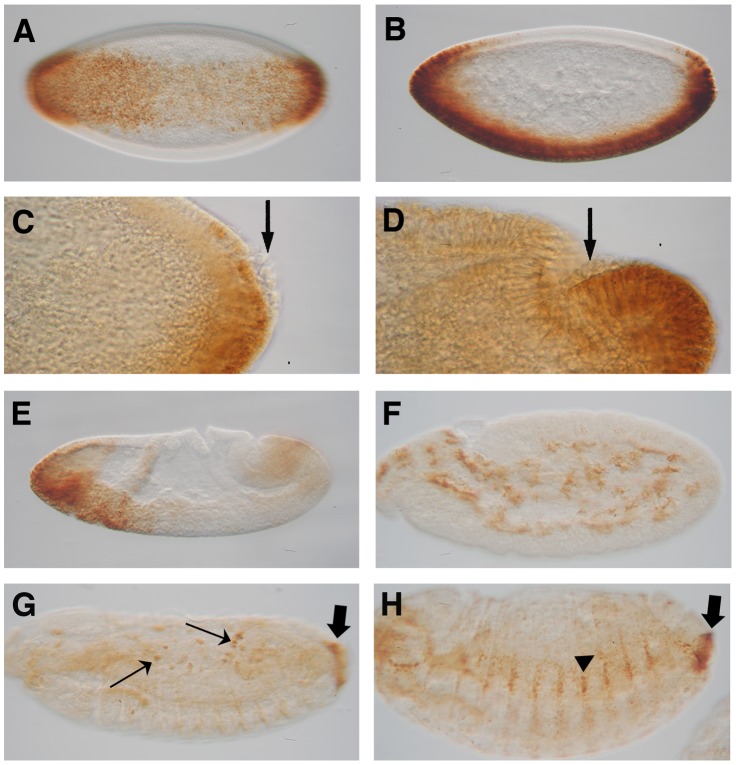
*Ndae1* expression in early embryogenesis. *Ndae1* expression patterns in embryos detected by *in situ* hybridization with TSA amplification followed by histochemistry (same as in figures 2 and 3). (A) Ventral expression in a ventral view of a stage 5 embryo. (B) Ventral expression in a lateral view of a stage 5 embryo. (C) Lateral view of the posterior end of a stage 5 embryo. *Ndae1* is not expressed in pole cells (arrow). (D) Lateral view of the posterior end of a stage 6 embryo. No *Ndae1* transcripts are observed in pole cells (arrow). (E) Lateral view of a gastrulating stage 6 embryo showing strong expression in the head and weak in the tail. (F) Lateral view of a stage 10 embryo. *Ndae1* is likely expressed in yolk cells. (G) Stage 12 embryo with amnioserosa expression (arrows) and the beginning of anal pad expression (block arrow). (H) Lateral view of a stage 14 embryo showing expression in lateral cells (arrowhead) and in the anal pads (block arrow).

**Figure 2:**
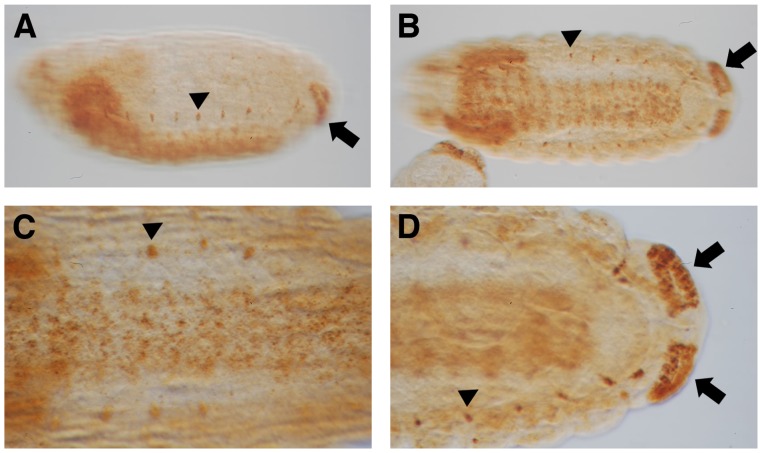
*Ndae1* expression in stage 15–16 embryos. (A) Lateral view of a stage 15 embryo showing expression in the CNS, in lateral cells and in the anal pads. (B) Ventral view of a stage 16 embryo expressing *Ndae1* in the CNS, in lateral cells and in the anal pads. (C) Magnification of a ventral view of a stage 16 embryo expressing *Ndae1* in the CNS and in lateral cells. (D) Magnification of the posterior end of a stage 16 embryo showing strong expression in the anal pads. Arrowheads: lateral cells. Block arrows: anal pads.

Previously, *Ndae1* transcripts have been detected by RT-PCR in embryos, larvae, pupae and adults [14]. In embryos, RNA was localized to the gut primordium, the mesoderm, the central nervous system (CNS) and the anal pads [14]. Subsequently, *Ndae1* has been detected by tyramide-ampified FISH in the heart [32]. Using polyclonal antibodies, *Ndae1* protein was found to be present at blastoderm stages, in the mesectoderm, the alimentary tract (gut and Malpighian tubules), the central nervous system (CNS), the peripheral nervous system (PNS), the head sensilla and the anal pads [18]. In order to clear the discrepancies between *Ndae1* transcript and protein localization, we performed *in situ* hybridization using a digoxigenin (DIG) labeled probe followed by tyramide amplification and revealing the staining by histochemistry (see Materials and Methods and Supporting Information). We found that *Ndae1* transcripts clearly begin to be expressed at the cellular blastoderm stage (stage 5), in a ventral band that extends dorsally at the anterior and posterior ends of the embryo (Figure 1B). *Ndae1* does not seem to be expressed in pole cells (Figures 1C and 1D, arrows) as instead previously reported [18]. At the beginning of gastrulation, *Ndae1* transcripts are observed in the head and weakly in the tail (Figure 1E). At germ band extension, *Ndae1* is detected in numerous cells that resemble yolk nuclei (vitellophages) (Figure 1F and see also http://insitu.fruitfly.org/cgi-bin/ex/report.pl?ftype=1&ftext=CG4675). At stage 13, expression in few amnioserosa cells is detected (Figure 1G, arrows) and *Ndae1* begins to be expressed in the anal pads (Figures 1G and 1H, block arrows). At stage 14, lateral cells are labeled (Figure 1H, arrowhead). At stage 15, transcripts are detected in the CNS, in the brain and in lateral clusters of cells with a segmented pattern (Figures 2A–D, arrowheads). Expression in the anal pads (block arrows in figures 1 and 2) persists until the end of embryogenesis and could potentially contribute to the mechanisms of osmoregulation and salt stress tolerance. No expression is detected with the sense strand except for salivary glands (data not shown and see [33]). We did not detect RNA expression in the mesectoderm, in the gut, in the Malpighian tubules and in the heart. This discrepancy could be due to the time window of expression of the transcripts in those tissues, which might be very narrow as *Ndae1* expression changes very rapidly. However, in spite of the differences in the detection methods used (TSA-amplified *in situ versus* traditional *in situ*), our results comply with those found by BDGP (http://insitu.fruitfly.org/cgi-bin/ex/report.pl?ftype=1&ftext=CG4675), despite few differences. Thus, our data reinforce the confidence in high throughput analysis of gene expression patterns in *Drosophila* that are publicly available while providing important complementary information.

**Figure 3:**
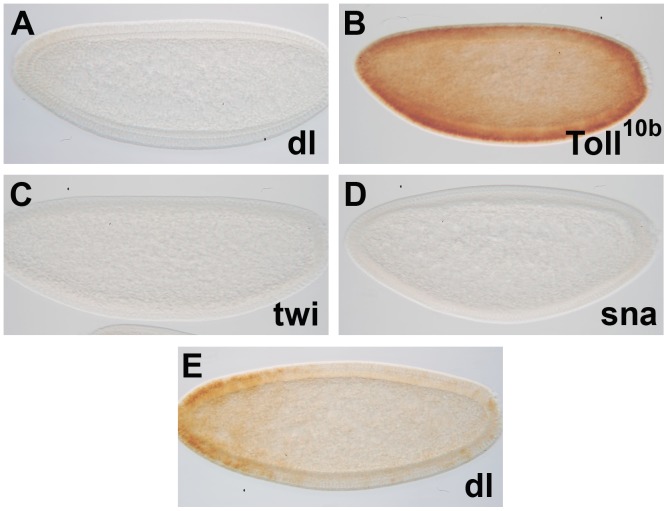
*Ndae1* expression in mutants of the DV pathway. (A) *dl^1^/dl^4^* embryo. (B) *Tl^10B^* embryo. (C) *twi^1^* embryo. (D) *sna^18^* embryo. *Ndae1* is not expressed in *dl*, *twi* and *sna* mutants, while it is expressed ectopically in *Tl* mutants. (E) Overstained *dl^1^/dl^4^* embryo in which residual anterior expression is observed. All embryos are at cellular blastoderm (stage 5).

Establishment of DV asymmetry in the early *Drosophila* embryo is largely under maternal control [35]–[38]. In the oocyte, *the dl* morphogen is distributed in the cytoplasm. Upon fertilization, activation of the *Tl* pathway is triggered when the maternal ligand *spaztle* binds to the transmembrane receptor *Tl* on the ventral side of the embryo [39]–[41]. Ultimately, *Tl* signaling leads to the nuclear localization of the *dl* morphogen specifically on the ventral side [42]–[44]. This is the initial cue for DV axis formation in the embryo. We investigated whether this pathway regulates the early ventral expression of *Ndae1*. In the majority (approximately 80%) of embryos from *dl^1^/dl^4^* transheterozygous mothers, no *Ndae1* expression is observed (Figure 3A compared with figures 1A and 1B). Consistently, in embryos from *Tl^10B^* mothers, where the *Tl* receptor is constitutively active, *Ndae1* expression extends dorsally (Figure 3B) emphasizing the role of the DV pathway in the control of early *Ndae1* expression. Noticeably, in few overstained *dl^1^/dl^4^* embryos some residual expression is observed anteriorly (Figure 3E), suggesting that *Ndae1* is also under the control of the anterior and/or the terminal pathways.

Due to the activation of the *Tl* receptor, Dl nuclear localization is highest in the ventral region. The activation of *dl* target genes occurs in a concentration dependent manner and has been subdivided in three categories (reviewed in [38], [45], [46]). *twi* and *sna* are the first two zygotic genes expressed in the ventral regions of the embryo in a domain where the levels of nuclear Dl are highest [47]–[49]. During gastrulation, *dl, twi* and *sna* cooperate to pattern ventral cell invagination to form the mesoderm [50], [51]. Mutants for either *twi* or *sna* fail to undergo a ventral invagination and lack mesodermal tissue. As *Ndae1* transcripts are expressed ventrally until gastrulation (Figures 1A and B), we explored *Ndae1* expression in *twi* and *sna* mutants. In *twi* (Figure 3C) or *sna* (Figure 3D) mutant embryos, no *Ndae1* expression is detected. Accordingly, we observed that in *twi, sna* double mutants *Ndae1* transcripts are also not present (data not shown). Altogether these data indicate that *Ndae1* is under control of the classical DV patterning cascade, in agreement with high throughput studies attempting to establish clusters of co-expressed genes at blastoderm stages, where *Ndae1* was predicted to be co-expressed with *sna* and *twi* [3].

**Figure 4:**
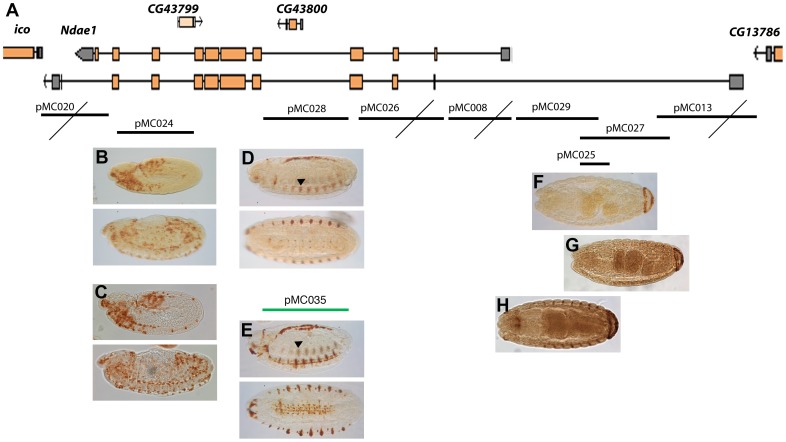
*Cis-*regulatory sequence analysis of *Ndae1*. (A) Genomic region of *Ndae1* (2L:7223328..7249343 in the R5.50 *Drosophila* genome sequence). Two of the nine splicing variants of *Ndae1* transcripts are represented. Genomic fragments cloned in reporter constructs are indicated by black or green lines corresponding to *lacZ* or *GFP* reporter constructs, respectively. Below each fragment there are representative stained embryos. Crossed lines indicate those fragments that do not drive any expression. Reporter constructs expression patterns are detected by immunohistochemistry. (B, C) β-Galactosidase (B) and lacZ mRNA (C) expression directed by pMC024 in rapidly changing cells from stage 10 until stage 15, likely being hemocytes. pMC028 (D) and pMC035 (E) drive expression in the CNS, in lateral cells (arrowheads) and in a dorsal line of amnioserosa cells (see figure 5). Fragments pMC025 (F), pMC027 (G) and pMC029 (H) drive the same expression pattern in the anal pads.

To fully understand animal transcription networks and predict cell fate, it is essential to measure the 3D spatial and temporal expression patterns of TFs and their downstream effectors and accurately map the *cis-*regulatory sequences of these TF targets [1], [52]. To this purpose, *Ndae1* is an appealing candidate because it is a downstream effector gene with a highly dynamic expression pattern during development. From a different point of view, the study of the *cis-*regulatory sequences of *Ndae1* might provide insights on its expression pattern discrepancies. Thus, we decided to study *Ndae1* enhancers through traditional methods. We first made *lacZ* reporter constructs covering the whole genomic region of *Ndae1* from the previous gene (*CG13786*) to the next one (*iconoclast*) (Figure 4). Using immunohistochemistry, we observed that four out of nine reporter constructs do not drive any expression (Figure 4A, crossed constructs). We found that construct pMC024 (Figure 4B) drives *lacZ* expression in the hemocytes, thus it probably belongs to a different gene. To better investigate the activity of this enhancer, we carried out *in situ* hybridization to detect *lacZ* mRNA in pMC024 embryos (Figure 4C). Protein and mRNA expression appear very similar. Construct pMC028, covering the fourth *Ndae1* intron, drives *lacZ* expression in the CNS (pMC028, Figure 4D), a domain of expression of *Ndae1* (Figure 2). *Ndae1* is one of the few ion exchangers with known functions in the control of cellular pH that have been described in the nervous system of *Drosophila* and pMC028 is a rare ion exchanger enhancer. We hope that in the future it will be a useful tool for studies on transcription control, specification in cells of the nervous system and network excitability. It would be interesting to understand whether the concentration of *Ndae1* is altered upon an action potential and whether the neuronal/glial selective loss of *Ndae1* affects excitability.

**Figure 5:**
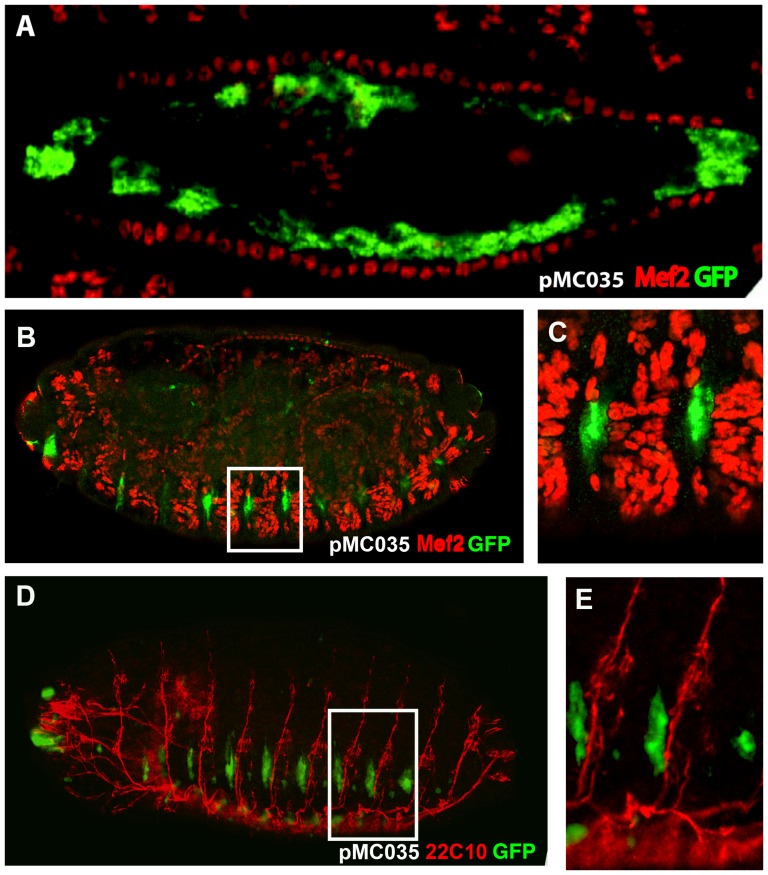
GFP expression driven by the fourth *Ndae1* intron. All embryos carry the pMC035 reporter construct and are stained by immunofluorescence with anti-GFP antibodies (green). (A) Magnification of a dorsal view of a stage 14 embryo double-stained with anti-Mef2 antibodies (red), labeling muscle cells including the cardiac tube. GFP is expressed in amnioserosa cells. (B) Lateral view of a stage 16 embryo double labeled with anti Mef-2 antibodies (red). (C) Magnification of a boxed portion of embryo in (B). The GFP-positive cells do not overlap the Mef-2-positive cells. (D) Lateral view of a stage 16 embryo double labeled with 22C10 antibodies (red). (E) Magnification of a boxed portion of embryo in (D). The GFP-positive cells lay below the 22C10-positive neurons.

Construct pMC028 drives expression also in a dorsal line of cells. In order to determine their belonging, we cloned the same intronic fragment into the pH-Stinger vector [31] (pMC035; Figure 4E) and observed that it drives GFP expression in the same domains as the *lacZ* construct, but with a higher signal. To determine the nature of these dorsal cells we performed double labeling with anti-Mef2 antibodies, which label all mesodermal cells including those of the heart where expression of *Ndae1* has been detected. We observed that the dorsal GFP-positive cells are large, polygonal and internal to the cardiac cells labeled by Mef2 (Figure 5A). Hence, we conclude they are amnioserosa cells. The amnioserosa is an extraembryonic epithelium that plays a key role in dorsal closure [53], the process by which the two dorsal epidermal margins, one on each side of the embryo, meet and fuse at the dorsal midline of the embryo at the end of embryogenesis. Inside the amnioserosa sheet, the external line of cells making direct contact with the dorsal epidermis has been described to have distinctive characteristics and plays a major role in the integrin-dependent adhesion of the amnioserosa cells to the dorsal epidermis in response to Dpp signaling [54]–[56]. We propose that the cells labeled by the reporter construct pMC035 (Figure 5A) belong to the peripheral or marginal amnioserosa group of cells, playing a role in germ band retraction [57]. This expression pattern is in agreement with previous proposals that *Ndae1* may contribute to baso-lateral ion transport in *Drosophila* epithelia. Expression of *Ndae1* in these cells might be too weak or transient to be detected even by enhanced *in situ* hybridization.

Both pMC028 and pMC035 constructs label also lateral cells (Figures 4D and 4E) that resemble a domain of expression of *Ndae1* (arrowheads in Figures 1H and 2). To learn more about the identity of these lateral cells we stained pMC035 embryos with anti-GFP and anti-22C10 antibodies (Figures 5B and 5C), which at the end of embryogenesis label several CNS neurons, PNS neurons and sensory neurons [58]. However, at dorsal closure, these lateral cells were not labeled with 22C10, but are interestingly in very close apposition, immediately below the 22C10-positive cells (Figure 5B). We also found that these cells do not belong to the mesoderm, as they are not labeled by anti-Mef2 antibodies (Figures 5D and 5E).

Moreover, three overlapping constructs in the 5′ region (pMC025, pMC027 and pMC029) drive expression in the anal pads (Figures 4F-H), a domain of expression of *Ndae1* (Figure 2). Because the anal pads are involved in osmoregulation [59]–[61], this enhancer may be very useful to study the contribution of *Ndae1* to osmotic balance in *Drosophila* and its role in the stress response due to changes in osmolarity of the medium.

Finally, none of the constructs reported expression at early blastoderm stages. We conclude that this blastoderm enhancer is probably lying in a genomic region far from the gene itself. We have not found any enhancer even for the pole cells, mesectoderm, Malpighian tubules, gut and heart where *Ndae1* had been previously described to be present [18], [32], nor did we detect *Ndae1* expression in these tissues. This could be due to the different techniques used or to a high instability of the *Ndae1* mRNA.

## Conclusions

Altogether, the data presented here indicate that *Ndae1* is under tight control during development. Some expression domains previously found have not been validated by our sensitive *in situ* hybridization procedure. We have revealed that the ventral blastoderm expression of *Ndae1* is regulated directly or indirectly by the classical DV pathway through *dl*, *Toll*, *twi* and *sna,* which had not been previously reported. Moreover, we have presented a sequential cloning of the whole genomic region of *Ndae1* in order to reveal all the areas positive for enhancer activity. The *Ndae1* enhancers found, especially the anal pads and CNS ones, provide new tools to validate future studies of *cis-*regulatory sequence prediction at a genome wide scale and to study *Ndae1* function. The different requirements of *Ndae1* in different neuron and/or glia adult subtypes as well as its role in network excitability in embryos and adult flies should be further explored. In addition, it would be interesting to understand whether *Ndae1* is altered in fly models for epilepsy or neurodegenerative diseases.

## Supporting Information

Text S1 Detailed protocol to carry out *in situ* hybridization with tyramide amplification on *Drosophila* embryos.
(PDF)

## References

[1] Fowlkes CC, Hendriks CLL, Keränen SVE, Weber GH, Rübel O, et al (2008) A quantitative spatiotemporal atlas of gene expression in the Drosophila blastoderm. Cell 133: 364–374 10.1016/j.cell.2008.01.053 18423206

[2] Segal E, Raveh-Sadka T, Schroeder M, Unnerstall U, Gaul U (2008) Predicting expression patterns from regulatory sequence in Drosophila segmentation. Nature 451: 535–540 10.1038/nature06496 18172436

[3] Frise E, Hammonds AS, Celniker SE (2010) Systematic image-driven analysis of the spatial Drosophila embryonic expression landscape. Mol Syst Biol 6: 345 10.1038/msb.2009.102 20087342 PMC2824522

[4] Buecker C, Wysocka J (2012) Enhancers as information integration hubs in development: lessons from genomics. Trends in Genetics 28: 276–284 10.1016/j.tig.2012.02.008 22487374 PMC5064438

[5] Naval Sanchez M, Potier D, Haagen L, Sánchez M, Munck S, et al (2013) Comparative motif discovery combined with comparative transcriptomics yields accurate targetome and enhancer predictions. Genome Res 23: 74–88 10.1101/gr.140426.112 23070853 PMC3530685

[6] Herrmann C, Van de Sande B, Potier D, Aerts S (2012) i-cisTarget: an integrative genomics method for the prediction of regulatory features and cis-regulatory modules. Nucleic acids research 40: e114 10.1093/nar/gks543 22718975 PMC3424583

[7] Ganetzky B (2000) Genetic analysis of ion channel dysfunction in Drosophila. Kidney Int 57: 766–771 10.1046/j.1523-1755.2000.00913.x 10720927

[8] Marban E (2002) Cardiac channelopathies. Nature 415: 213–218. 11805845 10.1038/415213a

[9] Cerrone M, Napolitano C, Priori SG (2012) Genetics of ion-channel disorders. Current opinion in cardiology 27: 242–252 10.1097/HCO.0b013e328352429d 22450718

[10] Créton R, Kreiling JA, Jaffe LF (2000) Presence and roles of calcium gradients along the dorsal-ventral axis in Drosophila embryos. Dev Biol 217: 375–385 10.1006/dbio.1999.9542 10625561

[11] Fontenele M, Carneiro K, Agrellos R, Oliveira D, Oliveira-Silva A, et al (2009) The Ca2+-dependent protease Calpain A regulates Cactus/I kappaB levels during Drosophila development in response to maternal Dpp signals. Mech Dev 126: 737–751 10.1016/j.mod.2009.04.005 19442719

[12] Pym ECG, Southall TD, Mee CJ, Brand AH, Baines RA (2006) The homeobox transcription factor Even-skipped regulates acquisition of electrical properties in Drosophila neurons. Neural Dev 1: 3 10.1186/1749-8104-1-3 17147779 PMC1679800

[13] Wolfram V, Southall TD, Brand AH, Baines RA (2012) The LIM-homeodomain protein islet dictates motor neuron electrical properties by regulating K(+) channel expression. Neuron 75: 663–674 10.1016/j.neuron.2012.06.015 22920257 PMC3427859

[14] Romero MF, Henry D, Nelson S, Harte PJ, Dillon AK, et al (2000) Cloning and characterization of a Na+-driven anion exchanger (NDAE1). A new bicarbonate transporter. J Biol Chem 275: 24552–24559 10.1074/jbc.M003476200 10827195

[15] Romero MF, Fulton CM, Boron WF (2004) The SLC4 family of HCO 3 - transporters. Pflugers Arch 447: 495–509 10.1007/s00424-003-1180-2 14722772

[16] Romero MF, Chen A-P, Parker MD, Boron WF (2013) The SLC4 family of bicarbonate (HCO^−^) transporters. Mol Aspects Med 34: 159–182 10.1016/j.mam.2012.10.008 23506864 PMC3605756

[17] FitzHarris G, Baltz JM (2009) Regulation of intracellular pH during oocyte growth and maturation in mammals. Reproduction 138: 619–627 10.1530/REP-09-0112 19520797

[18] Sciortino CM, Shrode LD, Fletcher BR, Harte PJ, Romero MF (2001) Localization of endogenous and recombinant Na(+)-driven anion exchanger protein NDAE1 from Drosophila melanogaster. Am J Physiol Cell Physiol 281: C449–C463. 11443044 10.1152/ajpcell.2001.281.2.C449

[19] Soleimani M, Burnham CE (2000) Physiologic and molecular aspects of the Na+:HCO3- cotransporter in health and disease processes. Kidney Int 57: 371–384 10.1046/j.1523-1755.2000.00857.x 10652014

[20] Cordat E, Casey JR (2009) Bicarbonate transport in cell physiology and disease. Biochem J 417: 423–439 10.1042/BJ20081634 19099540

[21] Parker MD, Qin X, Williamson RC, Toye AM, Boron WF (2012) HCO(3)(-)-independent conductance with a mutant Na(+)/HCO(3)(-) cotransporter (SLC4A4) in a case of proximal renal tubular acidosis with hypokalaemic paralysis. J Physiol (Lond) 590: 2009–2034 10.1113/jphysiol.2011.224733 22331414 PMC3573318

[22] Chesler M (2003) Regulation and modulation of pH in the brain. Physiol Rev 83: 1183–1221 10.1152/physrev.00010.2003 14506304

[23] Sinning A, Liebmann L, Kougioumtzes A, Westermann M, Bruehl C, et al (2011) Synaptic glutamate release is modulated by the Na+ -driven Cl-/HCO^−^ exchanger Slc4a8. J Neurosci 31: 7300–7311 10.1523/JNEUROSCI.0269-11.2011 21593314 PMC6622604

[24] Jacobs S, Ruusuvuori E, Sipilä ST, Haapanen A, Damkier HH, et al (2008) Mice with targeted Slc4a10 gene disruption have small brain ventricles and show reduced neuronal excitability. Proc Natl Acad Sci U S A 105: 311–316 10.1073/pnas.0705487105 18165320 PMC2224208

[25] Gurnett CA, Veile R, Zempel J, Blackburn L, Lovett M, et al (2008) Disruption of sodium bicarbonate transporter SLC4A10 in a patient with complex partial epilepsy and mental retardation. Arch Neurol 65: 550–553 10.1001/archneur.65.4.550 18413482

[26] Krepischi ACV, Knijnenburg J, Bertola DR, Kim CA, Pearson PL, et al (2010) Two distinct regions in 2q24.2-q24.3 associated with idiopathic epilepsy. Epilepsia 51: 2457–2460 10.1111/j.1528-1167.2010.02742.x 21204806

[27] Sebat J, Lakshmi B, Malhotra D, Troge J, Lese-Martin C, et al (2007) Strong association of de novo copy number mutations with autism. Science 316: 445–449 10.1126/science.1138659 17363630 PMC2993504

[28] Devinsky O, Vezzani A, Najjar S, De Lanerolle NC, Rogawski MA (2013) Glia and epilepsy: excitability and inflammation. Trends Neurosci 36: 174–184 10.1016/j.tins.2012.11.008 23298414

[29] Vossel KA, Beagle AJ, Rabinovici GD, Shu H, Lee SE, et al (2013) Seizures and epileptiform activity in the early stages of Alzheimer disease. JAMA Neurol 70: 1158–1166 10.1001/jamaneurol.2013.136 23835471 PMC4013391

[30] Winkelmann A, Maggio N, Eller J, Caliskan G, Semtner M, et al. (2014) Changes in neural network homeostasis trigger neuropsychiatric symptoms. J Clin Invest. doi:10.1172/JCI71472. PMC3904623 24430185

[31] Barolo S, Carver L, Posakony J (2000) GFP and beta-galactosidase transformation vectors for promoter/enhancer analysis in Drosophila. Biotechniques 29: 726, 728, 730, 732. 10.2144/00294bm10 11056799

[32] Perrin L, Monier B, Ponzielli R, Astier M, Sémériva M (2004) Drosophila cardiac tube organogenesis requires multiple phases of Hox activity. Dev Biol 272: 419–431 10.1016/j.ydbio.2004.04.036 15282158

[33] Amodio V, Tevy MF, Traina C, Ghosh TK, Capovilla M (2012) Transactivation in Drosophila of human enhancers by human transcription factors involved in congenital heart diseases. Developmental dynamics: an official publication of the American Association of Anatomists 241: 190–199 10.1002/dvdy.22763 21990232 PMC3326377

[34] Bernardoni R, Kammerer M, Vonesch J-L, Giangrande A (1999) Gliogenesis Depends on glide/gcm through Asymmetric Division of Neuroglioblasts. Developmental biology 216: 265–275 10.1006/dbio.1999.9511 10588877

[35] Morisato D, Anderson KV (1995) Signaling pathways that establish the dorsal-ventral pattern of the Drosophila embryo. Annu Rev Genet 29: 371–399 10.1146/annurev.ge.29.120195.002103 8825480

[36] Belvin MP, Anderson KV (1996) A conserved signaling pathway: the Drosophila toll-dorsal pathway. Annu Rev Cell Dev Biol 12: 393–416 10.1146/annurev.cellbio.12.1.393 8970732

[37] Moussian B, Roth S (2005) Dorsoventral axis formation in the Drosophila embryo—shaping and transducing a morphogen gradient. Curr Biol 15: R887–R899 10.1016/j.cub.2005.10.026 16271864

[38] Reeves GT, Stathopoulos A (2009) Graded dorsal and differential gene regulation in the Drosophila embryo. Cold Spring Harb Perspect Biol 1: a000836 10.1101/cshperspect.a000836 20066095 PMC2773625

[39] Schneider DS, Hudson KL, Lin TY, Anderson KV (1991) Dominant and recessive mutations define functional domains of Toll, a transmembrane protein required for dorsal-ventral polarity in the Drosophila embryo. Genes Dev 5: 797–807. 1827421 10.1101/gad.5.5.797

[40] Morisato D, Anderson KV (1994) The spätzle gene encodes a component of the extracellular signaling pathway establishing the dorsal-ventral pattern of the Drosophila embryo. Cell 76: 677–688. 8124709 10.1016/0092-8674(94)90507-x

[41] Winans KA, Hashimoto C (1995) Ventralization of the Drosophila embryo by deletion of extracellular leucine-rich repeats in the Toll protein. Mol Biol Cell 6: 587–596. 7663024 10.1091/mbc.6.5.587 PMC301217

[42] Roth S, Stein D, Nüsslein-Volhard C (1989) A gradient of nuclear localization of the dorsal protein determines dorsoventral pattern in the Drosophila embryo. Cell 59: 1189–1202. 2688897 10.1016/0092-8674(89)90774-5

[43] Rushlow CA, Han K, Manley JL, Levine M (1989) The graded distribution of the dorsal morphogen is initiated by selective nuclear transport in Drosophila. Cell 59: 1165–1177. 2598265 10.1016/0092-8674(89)90772-1

[44] Steward R (1989) Relocalization of the dorsal protein from the cytoplasm to the nucleus correlates with its function. Cell 59: 1179–1188. 2598266 10.1016/0092-8674(89)90773-3

[45] Stathopoulos A, Levine M (2005) Genomic regulatory networks and animal development. Dev Cell 9: 449–462 10.1016/j.devcel.2005.09.005 16198288

[46] Hong J-W, Hendrix DA, Papatsenko D, Levine MS (2008) How the Dorsal gradient works: insights from postgenome technologies. Proc Natl Acad Sci U S A 105: 20072–20076 10.1073/pnas.0806476105 19104040 PMC2629255

[47] Jiang J, Kosman D, Ip YT, Levine M (1991) The dorsal morphogen gradient regulates the mesoderm determinant twist in early Drosophila embryos. Genes Dev 5: 1881–1891. 1655572 10.1101/gad.5.10.1881

[48] Ip YT, Park RE, Kosman D, Yazdanbakhsh K, Levine M (1992) dorsal-twist interactions establish snail expression in the presumptive mesoderm of the Drosophila embryo. Genes Dev 6: 1518–1530. 1644293 10.1101/gad.6.8.1518

[49] Jiang J, Levine M (1993) Binding affinities and cooperative interactions with bHLH activators delimit threshold responses to the dorsal gradient morphogen. Cell 72: 741–752. 8453668 10.1016/0092-8674(93)90402-c

[50] Ip YT, Gridley T (2002) Cell movements during gastrulation: snail dependent and independent pathways. Curr Opin Genet Dev 12: 423–429. 12100887 10.1016/s0959-437x(02)00320-9

[51] Stathopoulos A, Van Drenth M, Erives A, Markstein M, Levine M (2002) Whole-genome analysis of dorsal-ventral patterning in the Drosophila embryo. Cell 111: 687–701. 12464180 10.1016/s0092-8674(02)01087-5

[52] Kazemian M, Blatti C, Richards A, McCutchan M, Wakabayashi-Ito N, et al. (2010) Quantitative analysis of the Drosophila segmentation regulatory network using pattern generating potentials. PLoS Biol 8. doi:10.1371/journal.pbio.1000456. PMC2923081 20808951

[53] Scuderi A, Letsou A (2005) Amnioserosa is required for dorsal closure in Drosophila. Dev Dyn 232: 791–800 10.1002/dvdy.20306 15704109

[54] Narasimha M, Brown NH (2004) Novel functions for integrins in epithelial morphogenesis. Curr Biol 14: 381–385 10.1016/j.cub.2004.02.033 15028212

[55] Fernández BG, Arias AM, Jacinto A (2007) Dpp signalling orchestrates dorsal closure by regulating cell shape changes both in the amnioserosa and in the epidermis. Mech Dev 124: 884–897 10.1016/j.mod.2007.09.002 17950580

[56] Wada A, Kato K, Uwo MF, Yonemura S, Hayashi S (2007) Specialized extraembryonic cells connect embryonic and extraembryonic epidermis in response to Dpp during dorsal closure in Drosophila. Dev Biol 301: 340–349 10.1016/j.ydbio.2006.09.020 17034783

[57] Lamka ML, Lipshitz HD (1999) Role of the amnioserosa in germ band retraction of the Drosophila melanogaster embryo. Dev Biol 214: 102–112 10.1006/dbio.1999.9409 10491260

[58] Hummel T, Krukkert K, Roos J, Davis G, Klämbt C (2000) Drosophila Futsch/22C10 is a MAP1B-like protein required for dendritic and axonal development. Neuron 26: 357–370. 10839355 10.1016/s0896-6273(00)81169-1

[59] Jarial MS (1987) Ultrastructure of the anal organ of Drosophila larva with reference to ion transport. Tissue Cell 19: 559–575. 18620212 10.1016/0040-8166(87)90048-6

[60] Keyser P, Borge-Renberg K, Hultmark D (2007) The Drosophila NFAT homolog is involved in salt stress tolerance. Insect Biochem Mol Biol 37: 356–362 10.1016/j.ibmb.2006.12.009 17368199

[61] Mehta A, Deshpande A, Missirlis F (2008) Genetic screening for novel Drosophila mutants with discrepancies in iron metabolism. Biochem Soc Trans 36: 1313–1316 10.1042/BST0361313 19021547

