# Supplementary material for: Ndae1 Expression and Regulation in Drosophila Embryos
Source: PLoS One. 2014 Mar 27;9(3):e92956. doi: 10.1371/journal.pone.0092956 (PMC3968038; doi:10.1371/journal.pone.0092956)
Supplement: Text S1 — Detailed protocol to carry out in situ hybridization with tyramide amplification on Drosophila embryos. (PDF) [file pone.0092956.s001.pdf]

Supplementary Information Text S1

Ndae1 expression and regulation in _Drosophila_ embryos

Maria Florencia Tevy, Denis Seyres, Concetta Traina, Laurent Perrin and Maria Capovilla

WHOLE MOUNT *IN SITU* HYBRIDIZATION ON *DROSOPHILA* EMBRYOS WITH TSA

by Maria Capovilla

EMBRYO FIXATION

1) *Collection.* Put collection basket (made with falcon tubes and nylon mesh) in water. Collect embryos from plate with a paint brush and transfer in basket.

2) *Dechorionation.* Rinse embryos well with water and transfer basket in 50% bleach. Let sit for 2 minutes, swirling embryos around once in a while and adding bleach if needed. Rinse with water.

From (3) to (6) in hood!:

3) *Transfer.* Make all embryos go on mesh, blot basket on paper towel, remove mesh and immerse it in 800 µl Heptane (Sigma #H-9629) in an eppendorf using forceps. Embryos that stick to the mesh are old (have the cuticle), so it is better to throw them away.

4) *Fixation.* Add 300 µl of fix-buffer and 200 µl l of 10% methanol free formaldehyde (Polysciences #04018). Let rotate or shake gently for 30min (or at least 20min).

5) *Devitellinization.* Remove fixative (bottom phase) and dispose properly. Fill eppendorf with 100% Methanol. Shake vigorously or vortex for 15 sec. Most of the embryos should sink to the bottom of the tube. If not, shake/vortex some more.

6) *Storage.* Remove all liquid starting from interphase (contains vitelline membranes). Fill with 100% Ethanol, close and invert the tube. Let embryos settle, remove Ethanol and rinse 2 more times with 100% Ethanol. Store at 4°C (short term) or -20°C (long term and *in situ*).

❖ All washes are carried out in 1 ml.

EMBRYO TREATMENT AND POST-FIX

- Rehydrate embryos 1x5 min in 50%EtOH-50%PBT-DEPC.
- Wash 3x10min in PBT-DEPC (1xPBS + 0.1% Tween-20).
- OPTIONAL: inactivate endogenous HRP 30min in 3% H_2O_2 (100 µl 30% H_2O_2 in 900 µl PBT-DEPC). Wash 3x5min in PBT-DEPC.
- Post-fix and permeabilize embryos 30 min in 1 ml of 1xPBS + 4% Formaldehyde methanol-free + 0.8% Triton X-100 (prepared fresh each time).
- Wash 2x10min in PTX-DEPC (1xPBS + 0.3% Triton X-100).

HYBRIDIZATION

Test every new probe using different dilutions from the mother probe solution: 1/100, 1/1000, 1/5000 (the good dilution depending on the transcription efficacy). Sometimes, low signal is due to too concentrated probe.

- Wash 2x5min in PTX:HS 1:1 (no SS DNA).

During this time, prepare the needed amount of Hybridization Solution (HS) (1 ml/tube + amount for hybridization/tube) containing 100 µg/ml Salmon Sperm DNA and 0.5 mg/ml tRNA (or less). Boil SS DNA 5-10min, spin, mix well and add to HS. Mix and keep on ice.

- Wash 2x10min in HS (no SS DNA, no tRNA).
- Prehybridize in 0,5 ml HS (with SS DNA and tRNA) 2 x 30 min at 55°C. Mix once in a while.
- Heat probe (already in HS) at 75°C for 5-10min. Spin, mix well and put on ice.
- Add the appropriate amount of probe to the appropriate amount of HS + SS DNA + tRNA on ice (100 ng/sample or less if you use the TSA Plus kit).
- Remove prehybridization solution.
- Add the probe mixing very well the embryos with the blue tip or by flicking.
- Incubate O/N (12-16hrs) at 55°C.

POST-HYBRIDIZATION WASHES
IMPORTANT: *save probes at -20°C!* Sometimes re-used probes work very well.
Prepare and prewarm all the solutions before use.

Wash at 55°C:	2x20min in HS (no tRNA, no SS DNA)
	1x20min 3:1=HS:PTX
	1x20min 1:1=HS:PTX
	1x20min 1:3=HS:PTX
Wash at room temperature:	
	1x10min PTX
	2x10min TNT

BLOCK ENDOGENOUS BIOTIN (KIT VECTORLAB #SP2001)
- Incubate 15min in Avidin (one drop in 400 μl TNT).
- Wash 3x5min with TNT.
- Incubate 15min in Biotin (one drop in 400 μl TNT).
- Wash 3x5min in TNT.
- Block 3x10min in TNB.

ANTI-DIG ANTIBODY INCUBATION
- Incubate O/N in 250 μl of 1:250 preabsorbed Biotinylated anti-DIG (Jackson ImmunoResearch #200-062-156) in TNB.

TSA PROCEDURE
Reagents:
- kit TSA Plus Biotin System, Perkin Elmer n. NEL749A001KT or n. NEL749B001KT
- 1X Plus Amplification Diluent, Perkin Elmer n. FP1135 (Perkin Elmer does not like to sell it separately, but they can. I purchased it many times.)
- Streptavidin-HRP, Perkin Elmer n. NEL750001EA.

- Wash 2x10min in TNT.
- Block 3x10min in TNB (900 μl).
- Incubate 40 min in 250 μl of 1:500 SA-HRP in TNB.
- Wash 3x10 min in TNT.
- Transfer in 0.5 ml tube.
- Equilibrate 10 min in 100 μl Amplification Buffer.
- Dilute Biotinyl Tyramide Stock Solution 1:500 in 1x Amplification Diluent buffer. Use 250 μl/sample. It may be worth to test different dilutions for each probe.

- Perkin Elmer recommends 1:50. The higher the concentration, the more sensitive it is, but the higher the background.

- Incubate 15 min in Biotinyl Tyramide Working Solution (diluted TSA).
- Transfer to 1.5 ml tube.
- Wash 3x10 min in TNT.
- Block 3x10 min in TNB (900 µl).
- Incubate 40 min in 1:500 SA-HRP in TNB. Use 250 µl/sample.
- Wash 2x10min in TNT.
- Wash 3x10min in PBT.

STAINING

- Dilute 5 µl of 30% HYDROGEN PEROXIDE in 495 µl of H_2O.
- Mix:

Mix:	PBT	1 ml
	DAB (5mg/ml)	100 µl
	H_2O_2 (0.3%)	5-10 µl

Color should develop within 1-10min. Stop staining by rinsing with PBT.
NOTE: DAB is cancerogenic! Wear gloves and dispose properly.

MOUNTING

- Wash 1 x 5-10 min in 50% PBT-50% EtOH.
- Wash 2 x10min in 100% EtOH.
- You may stop at this point and store the embryos at 4°C.
- Clear embryos 5-10min in 800 µl of Methyl Salicylate (digests the yolk).
- Fill tube with 100% EtOH. Let embryos sit. Remove Methyl Salicylate as much as possible without removing embryos and fill tube with 100% EtOH. Mix.
- As soon as embryos settle, rinse 2 x 5 min with 100% EtOH.
- Mount embryos in Canada Balsam (SIGMA #C1795).

SOLUTIONS

FIXATION BUFFER (50 ml)

H_2O	25 ml
10 x PBS	5 ml
0.5M EGTA	100 µl
1M $MgSO_4$	100 µl

PBS
SIGMA-ALDRICH tablets n. P4417.

20 X SSC – Maniatis (1L)

NaCl	175.3 g
NaCitrate	88.2 g
dH_2O	up to 1000 ml

Adjust pH to 7.0 with 10N HCl.
Add 1ml DEPC/1000 ml, let sit O/N at 37°C and autoclave.

HYBRIDIZATION SOLUTION (HS) (200ml)

Formamide (deionized)	100 ml (50% final)
20 X SSC (DEPC)	50 ml (5X final)
50mg/ml Heparin	200 µl (50 µg/ml final)
Triton X-100	600 µl (0.3% final)
DEPC H_2O	50 ml

Prepare in DEPC-treated bottle.
Aliquot in 50ml Falcon, number and store at -20°C.
NOTE: formamide is sentisitive to light!

SALMON SPERM DNA (SS DNA)

- Prepare a 10mg/ml stock in ddH_2O in a 50 ml screw cap tube (not more than 40 ml per 50 ml tube).
- Let it dissolve O/N at 4°C.
- Boil 10-15min or until well resuspended.
- Separate in 20ml aliquots and sonicate.
- Combine and aliquot. Store at -20°C.
- Run 1 µg on a gel to verify its size.

PBT (1000 ml)

10 X PBS	100 ml
Tween - 20	1 ml (0.1% final)

PTX (500 ml)

10 X PBS	50 ml
Triton X-100	1.5 ml (0.3% final)

POST FIX SOLUTION (<u>1ml = 1 sample</u>)

DEPC dH_2O	500 µl
10 X PBS-DEPC	100 µl (1x final)
10% methanol-free Formaldehyde	400 µl (4% final)
Triton X-100	8 µl (0.8% final)

TNT (500 ml)

2M Tris pH7.5	25 ml (0.1M final)
5M NaCl	15 ml (0.15 M final)
Tween-20	250 µl (0.05% final)
dH_2O	460 ml

TNB (100 ml)

2M Tris pH7.5	5 ml (0.1 M final)
5M NaCl	3 ml (0.15M final)
Blocking Reagent (supplied in kit)	0.5 g (0.5%)
dH_2O	92 ml
